# Gestational changes in iodine status in a cohort study of pregnant women from the United Kingdom: season as an effect modifier^1^,^2^,^3^

**DOI:** 10.3945/ajcn.114.105536

**Published:** 2015-05-06

**Authors:** Sarah C Bath, Victoria L Furmidge-Owen, Christopher WG Redman, Margaret P Rayman

**Affiliations:** 1 From the University of Surrey, Guildford, United Kingdom (SCB, VF-O, and MPR); Danone Nutricia Early Life Nutrition, Trowbridge, United Kingdom (VF-O); and the Nuffield Department of Obstetrics and Gynecology, University of Oxford, Oxford, United Kingdom (CWGR).

**Keywords:** deficiency, diet, iodine, pregnancy, United Kingdom

## Abstract

**Background:** Iodine is required throughout pregnancy for thyroid hormone production, which is essential for fetal brain development. Studies of iodine status in pregnant women from the United Kingdom (UK) have focused on early gestation (<16 wk). Data on the effect of advancing gestation on urinary iodine excretion are conflicting, with suggestions of both an increase and a decrease.

**Objectives:** The aims were to evaluate iodine status in a cohort of UK pregnant women and to explore how it changes throughout gestation.

**Design:** We used samples and data from 230 UK pregnant women who were recruited to the Selenium in PRegnancy INTervention study. Iodine concentration was measured in spot-urine samples that were collected at ∼12, 20, and 35 wk of gestation; creatinine concentration was also measured to correct for urine dilution. A linear mixed model was used to explore the effect of gestational week on iodine-to-creatinine ratio, with change in season, body mass index, daily milk intake, and maternal age controlled for.

**Results:** The median urinary iodine concentration from urine samples collected at all time points (*n* = 662) was 56.8 μg/L, and the iodine-to-creatinine ratio was 116 μg/g, thus classifying this cohort as mildly-to-moderately iodine deficient. The median iodine-to-creatinine ratios at 12, 20, and 35 wk were 102.5, 120.0, and 126.0 μg/g, respectively. Only 3% of women were taking iodine-containing prenatal supplements. The iodine-to-creatinine ratio increased with advancing gestation, and there was a significant interaction between gestational week and season (*P* = 0.026). For a 1-wk increase in gestation, the iodine-to-creatinine ratio increased by a factor of 1.05 (95% CI: 1.02, 1.08) in winter and by a factor of 1.04 (95% CI: 1.00, 1.08) in summer.

**Conclusions:** This group of UK pregnant women was mildly-to-moderately iodine deficient at all trimesters, which is of public health concern. The finding that the iodine-to-creatinine ratio increased over the course of gestation may not be generalizable to populations with different iodine status from ours and merits further investigation. This trial was registered at www.isrctn.com as ISRCTN37927591.

## INTRODUCTION

Iodine is essential for the production of the thyroid hormones thyroxine and tri-iodothyronine. Because these are vital for brain and neurological development of the fetus during pregnancy and early life (1), iodine deficiency is of considerable public health concern. Indeed, iodine deficiency during pregnancy in regions of mild-to-moderate iodine deficiency has been associated with poorer intelligence quotient, reading scores (2), and spelling ability (3) in children up to the age of 9 y.

Population iodine status is commonly assessed by measuring urinary iodine concentration (UIC),^4^ which reflects recent intake (1). The WHO recommends comparing the median UIC from spot-urine samples against cutoffs for describing population iodine status; iodine deficiency in a population of pregnant women is defined as a median UIC <150 μg/L (4). The United Kingdom has long been considered to be iodine sufficient, although in recent years this assumption has been challenged and iodine deficiency has been observed in teenage schoolgirls (5) and pregnant women (2, 6–9). Borderline iodine status was recently found in a group of women of childbearing age, despite the fact that most of them were studying for a nutrition degree (10). The UK studies in pregnant women have focused on iodine status in early gestation, which is a critical time for adequate iodine supply for fetal brain development (11). However, an adequate maternal intake of iodine is vital throughout pregnancy for the synthesis of maternal thyroxine, which is transferred to the fetus in early gestation, and for iodine supply to the fetus when the fetal thyroid begins to function around midgestation (11).

The effect of advancing gestation on iodine status is poorly understood, and evidence has been found for both an increase and a decrease in measured urinary iodine excretion across gestation (12). Investigating the change in iodine excretion as gestation advances is particularly challenging in the United Kingdom, where iodine intake alters with season. This is due to the marked seasonal difference in iodine concentration in UK dairy produce, which is the principal source of dietary iodine (13), and results in a seasonal difference in iodine status that is highest in the winter (5). Because pregnancy spans seasons, it is vital that the underlying change in iodine exposure (i.e., from the changing iodine content of dairy products) is appropriately controlled for when exploring the change in iodine excretion over gestation.

To our knowledge, there are no published UK data on iodine status in mid-to-late pregnancy (i.e., after 16 wk) and no data on the effect of advancing gestation on iodine status in UK pregnant women. We therefore aimed to evaluate how the iodine-to-creatinine ratio changes over the course of pregnancy in a cohort of women who provided urine samples in each trimester. We hypothesized that women would be classified as iodine deficient according to WHO criteria and that any change in iodine excretion across gestation would largely be explained by change in season.

## METHODS

### Participants

This study used samples and data collected as part of the Selenium in Pregnancy INTervention (SPRINT) study, a double-blind, placebo-controlled, randomized trial (ISRCTN37927591) that investigated the effect of selenium supplementation on markers of risk of pre-eclampsia. The methods of SPRINT have been previously reported (14). Briefly, between July 2009 and June 2011, 230 primiparous women were recruited when attending for an ultrasound scan at 12–14 wk of gestation at the John Radcliffe Hospital, Oxford, United Kingdom. The sample size was determined to detect differences in biological markers of pre-eclampsia. Women were followed up twice in pregnancy, at ∼20 and 35 wk of gestation. Among other exclusion criteria (14), women were not eligible if they were current smokers, taking thyroid medication, or taking a selenium-containing supplement.

Because there was no significant difference in iodine status (urinary iodine-to-creatinine ratio) between the selenium and placebo groups at 12 (*P* = 0.08), 20 (*P* = 0.96), or 35 (*P* = 0.68) wk of gestation, for the purposes of this investigation, analysis was conducted in all participants regardless of treatment group. It is relevant to note that we previously showed that selenium treatment had no effect on thyroid function or prevalence of thyroid disorders in the SPRINT cohort overall (15).

This study was conducted according to the guidelines in the Declaration of Helsinki, and all procedures involving human subjects were approved by the Milton Keynes Research Ethics Committee (REC reference 08/H0603/46). A nonsubstantial amendment for additional laboratory measurements in stored samples was approved by the National Research Ethics Service Committee South Central–Berkshire (27 July 2011). Written informed consent was obtained from all subjects.

### Procedures

Women provided a spot-urine sample at each of the 3 study visits, at ∼12, 20, and 35 wk of gestation, and thus we had measures in the first, second, and third trimester. The samples at 12 and 20 wk were collected when the women were attending the hospital for an ultrasound scan (14). We calculated the actual gestational week of the urine sample on the basis of gestational week of recruitment (14) and the dates of urine collection. There is a pronounced seasonal difference between the iodine concentration of winter and summer milk, the former having a concentration approximately double that of the latter (16). Because this difference affects iodine status (5, 17), the urine samples were broadly grouped as being from summer (May to October) or winter (November to April). The seasons were defined on the basis of a previous study of iodine status and milk-iodine content in the United Kingdom (18).

A food-frequency questionnaire (FFQ) was administered at recruitment and was completed by 219 women (95.6% of the cohort). At the same time, clinical and demographic data, including weight and height (used to calculate BMI at 12 wk), date of birth (used to calculate maternal age at recruitment), smoking status (ex-smoker or nonsmoker), age at which education ceased, and occupation were recorded. Maternal occupation was used to code maternal social class according to the National Readership Demographic categories (19) and was collapsed into 2 groups: *1*) A and B (middle class and above) and *2*) C1 to D (lower middle class and below).

The 18-item FFQ was designed to capture information on selenium- and iodine-rich foods (not to estimate selenium or iodine intake) and its design was based on that used in the European Prospective Investigation into Cancer and Nutrition study (20). Data were collected on consumption of seafood (white fish, oily and shellfish, fish fingers, and fish roe), meat (beef, beefburgers, pork and lamb, bacon, ham, sausages, and corned beef), and poultry, Brazil nuts, offal (liver and liver products), dairy products (grouped as 1 item in the questionnaire), and milk. Data on egg consumption were not collected. In cases in which participants had not given a frequency for individual food items (*n* = 7), these foods were coded as being consumed “never or rarely.” The answers were converted to weekly portions, and for seafood and meat and poultry the portions were summed to give a total. Food items were then recoded to reflect high and low intakes (i.e., above and below the median). Milk intake was recoded to 3 categories (<140, 140–280, and >280 mL), which resulted in approximately even numbers in each group. For liver products and Brazil nuts, the participants were dichotomized into either “consumers” (any frequency of consumption) or “nonconsumers” (those who answered as “never/rarely” consuming the products) because of the low numbers of consumers.

### Laboratory analysis

Urine was stored at −80°C until analysis for iodine and creatinine concentrations. The analysis was conducted at the Trace Element Unit, Southampton General Hospital, by using a dynamic reaction cell inductively coupled plasma mass spectrometer (Sciex Perkin-Elmer). Samples were analyzed against a urine calibration curve with the addition of 0, 1, 2, 5, and 10 μmol iodine/L (potassium iodide; Fisher Chemicals). Samples for the calibration, test, and quality control were diluted 1:14 with diluent [0.3% ammonia (Fisher Chemicals), 0.001 mmol/L (NH4)_2_H_2_EDTA (Fluka Chemika), and 0.01 mol/L NH_4_H_2_PO_4_ (British Drug Houses, Poole)]. Rhodium was used as the internal standard (VWR International). Samples were measured in duplicate. We verified the accuracy of results with the certified reference material, Seronorm Trace Elements Urine (Nycomed Pharma; certified iodine content: 304 μg/L; range: 270–338 μg/L) and internal quality-control samples D (target value: 35.5 μg/L; range: 33–38 μg/L) and E (120 μg/L; range: 115–125 μg/L). The observed values were 318 μg/L (SD: 10.1; *n* = 23) for the certified reference material, 36.8 μg/L (SD: 1.3; *n* = 27) for sample D, and 118 μg/L (SD: 2.5; *n* = 24) for sample E. Within-run precision was 2.0% at 0.28 μmol/L, 1.3% at 0.94 μmol/L, and 1.0% at 2.5 μmol/L. Between-run precision was 3.6% at 0.28 μmol/L, 2.5% at 0.94 μmol/L, and 3.3% at 2.5 μmol/L. Urinary creatinine was determined by the UniCel DxC Synchron Clinical System Analyzer (Beckman Coulter) by the Jaffe rate method.

### Statistical analysis

The iodine status of the group was described by comparing the median UIC value to the WHO UIC cutoffs for iodine adequacy in pregnancy (4). However, because UIC values cannot be used for estimation of individual iodine status, we used urinary creatinine concentration to correct UIC for variable dilution among spot-urine samples; the iodine-to-creatinine ratio produces a better measure of individual iodine status, especially when the age and sex of the individual is taken into account (21–23). This is especially important in this cohort because SPRINT women were requested to attend the hospital with a full bladder for their 12-wk ultrasound scan and, as a result, some urine samples were very dilute (visual inspection revealed samples that seemed little different from water).

We report iodine status in 2 ways: as the iodine concentration (μg/L) and as the iodine-to-creatinine ratio (μg/g). To explore relations with participant characteristics (e.g., age) and dietary intake (estimated from the FFQ), we used the iodine-to-creatinine ratio.

For the purposes of describing the prevalence of deficiency, we used the computed Estimated Average Requirement (EAR) cutoff for estimated 24-h urinary iodine excretion (160 μg/d, assuming 90% excretion of the EAR of 180 μg/d), as in our previous study in UK pregnant women (8). The 24-h excretion of iodine was estimated by multiplying the iodine-to-creatinine ratio by the expected daily excretion of creatinine [which is 1.23 g/d (21) for our cohort of women aged 18–43 y, with no expected difference in total creatinine excretion between trimesters (24)].

Means and SDs or medians and IQRs are reported for variables that are normally or nonnormally distributed, respectively. Because the iodine-to-creatinine ratio was not normally distributed, it was transformed using the natural logarithm to enable the use of parametric tests. Correlations between iodine excretion measured at 12, 20, and 35 wk were examined by using the Pearson correlation. Independent *t* tests or 1-factor ANOVA was used to compare log-transformed data between 2 groups or >2 groups, respectively.

Multiple linear regression (using the general linear model) was used to evaluate associations between dietary or demographic factors and the (log-transformed) iodine-to-creatinine ratio at 12 wk of gestation. Variables that showed a relation (*P* < 0.20) with the iodine-to-creatinine ratio in the unadjusted analyses were entered into the model. We chose to only examine cross-sectional relations between iodine status and diet and demographic variables at 12 wk because the FFQ was administered at this time point.

A linear mixed model was used to explore the effect of advancing gestation on the (log) iodine-to-creatinine ratio after controlling for the underlying change in season as women progress through pregnancy as well as other potential confounders identified from the literature and univariate analyses (BMI, milk intake, and maternal age). We used the actual gestational week of the urine sample (calculated as above) instead of trimester (i.e., 1, 2, or 3) for our analysis because this yielded more information for each woman; the variable had a range of 9–36 wk.

A linear mixed model was necessary to make maximum use of the available information, because some subjects had missing data. Fixed-effect variables in the model included both time-varying (i.e., varying within a subject) and non–time-varying (i.e., fixed within a subject) variables. The time-varying variable in the model was season, a categorical variable with 2 levels (summer or winter). The non–time-varying variables were maternal age (range: 18–43 y), BMI measurement at 12 wk of gestation [range (in kg/m^2^): 17–39], and daily milk intake (<140, 140–280, and >280 mL). The model also included random effects with random coefficients at the subject level (i.e., intercept and gestational week). This required estimating 3 extra variables: 1 variance each for the 2 random coefficients plus a covariance term. All 3-way and 2-way interactions between variables were assessed against a 10% significance level by using the respective *P* values from adjusted (Wald) hypothesis tests in the table of regression coefficients. The hierarchy of effects was preserved, that is, if an interaction was included, so were its main effect components. The standardized residuals at levels 1 (within-subject) and 2 (between-subjects) were visually assessed for normality. We estimated the multivariable-adjusted geometric mean ratios of the iodine-to-creatinine ratio for a 1-wk increase in gestation.

We acknowledge that creatinine correction may be imperfect in the case of very dilute samples, and we therefore conducted a sensitivity analysis that excluded very dilute samples. We defined dilute samples using the biological monitoring criterion [i.e., creatinine concentration <0.3 g/L (25)]. Dilute urine samples were excluded from the linear mixed-model analysis to explore whether differences by gestational week might be the result of a dilution bias in the samples collected at ultrasound scans at 12 and 20 wk (women, at 12 wk in particular, were asked to attend with a full bladder), even after creatinine correction.

Unless otherwise stated, significance was set at *P* < 0.05. All analyses were conducted by using the Statistical Package for Social Sciences (version 21.0; SPSS), except for the linear mixed model, which was conducted in SAS (version 9.3; SAS Institute).

## RESULTS

One woman was recruited in error; she was taking thyroxine and was therefore excluded from analysis. None of the women reported having chronic renal disease. Urine samples were collected from 228, 222, and 212 women at ∼12, 20, and 35 wk of gestation, respectively. A total of 212 women provided 3 urine samples, and 222 women provided at least 2 urine samples. As previously reported (14), the mean (±SD) age of the women at recruitment was 30.73 y ± 4.18 y, mean BMI was 24.59 ± 3.99, 93% were Caucasian, the mean age at which woman left education was 20.79 y ± 2.97 y; all were nonsmokers, 32% of whom were ex-smokers. There were no missing cases for demographic variables, with the exception of age at which education ceased (*n* = 1).

In total, 662 urine samples were collected (from all women and at all time points). The median UIC and iodine-to-creatinine ratio of these 662 samples was 56.8 μg/L (IQR: 31.1–104 μg/L) and 116 μg/g (IQR: 77–177 μg/g), respectively; the group was therefore classified as mildly-to-moderately deficient according to WHO criteria (4, 26). The median estimated 24-h urinary iodine excretion, at 143 μg/24 h (IQR: 95–219 μg/24 h) was considerably below the value of 160 μg/24 h that we calculated to reflect the urinary excretion of the EAR for pregnancy (8); 55.7% (*n* = 369) of samples were below that cutoff.

**Table 1** presents the iodine results for each trimester, showing that the women were classified as iodine deficient at each point. There were significant, moderate correlations between iodine-to-creatinine ratio measured at 12 and 20 (*r* = 0.520, *P* < 0.001), 12 and 35 (*r* = 0.452, *P* < 0.001), and 20 and 35 (*r* = 0.418, *P* < 0.001) wk of gestation.

**TABLE 1:** Urinary iodine status by trimester of urine sample collection (iodine concentration, iodine-to-creatinine ratio, and estimated 24-h iodine excretion)

	First trimester (*n* = 228)	Second trimester (*n* = 222)	Third trimester (*n* = 212)
Gestational week of sample^1^	12 (9, 16)	20 (17, 23)	35 (30, 36)
Iodine concentration,^2,3^ μg/L	42.0 (24.5–84.8)	52.0 (30.9–103.3)	69.4 (31.1–104.3)
Iodine-to-creatinine ratio,^2,4^ μg/g	103 (67–167)	120 (80–185)	126 (84–183)
Estimated 24-h iodine excretion,^2,4^ μg/d	126 (83–206)	147 (100–228)	155 (104–225)

1 Values are medians; minimum, maximum in parentheses.

2 Values are medians; IQRs in parentheses.

3,4 Significantly different between trimesters (Kruskal-Wallis test): ^3^*P* < 0.001, ^4^*P* = 0.013.

Maternal age and iodine-to-creatinine ratio at 12 wk of gestation were positively, although weakly, correlated (*r* = 0.13, *P* = 0.04); there was no correlation between maternal age and urinary creatinine concentration at any point in pregnancy. There was also a weak, positive correlation between age at which education ceased and iodine-to-creatinine ratio (*r* = 0.15, *P* = 0.03). Ethnicity, social class, and smoking status (non- or ex-smokers) were not significantly related to iodine-to-creatinine ratio (**Table 2**).

**TABLE 2:** Predictors of urinary iodine-to-creatinine ratio measured at ∼12 wk gestation^1^

	*n*	Median (IQR)	*P*^2^	Adjusted *P*^3^
Demographic factors				
Smoking status				
Nonsmoker	155	104 (72–172)	0.16	0.30
Ex-smoker	73	102 (60–163)		
Ethnicity				
Caucasian	212	104 (67–170)	0.72	NA
Other	16	92 (67–147)		
Social class				
Middle and above	100	107 (69–182)	0.41	NA
Lower middle and below	128	102 (64–157)		
Dietary factors				
Daily milk intake				
<140 mL	61	72 (48–99)	<0.001	<0.001
140–280 mL	84	104 (76–175)		
>280 mL	73	150 (96–219)		
Dairy products				
≤1 portion/d	109	93 (64–163)	0.19	0.59
>1 portion/d	109	116 (72–175)		
Seafood				
<2 portions/wk	97	107 (69–173)	0.62	NA
≥2 portions/wk	121	99 (65–164)		
Meat/poultry				
<7 portions/wk	108	98 (65–162)	0.40	NA
≥7 portions/wk	110	107 (69–178)		
Iodized salt				
Nonconsumer	205	104 (68–172)	0.28	NA
Consumer	13	94 (66–127)		
Brazil nuts				
Nonconsumer	192	99 (65–164)	0.07	0.12
Consumer	26	129 (92–186)		
Liver				
Nonconsumer	181	102 (67–173)	0.98	NA
Consumer	37	107 (75–157)		

1 Values are iodine-to-creatinine ratio, μg/g. NA, not applicable.

2 *P* values from an independent *t* test (or 1-factor ANOVA in the case of milk intake) were conducted on log-transformed data.

3 Variables with *P* < 0.20 in unadjusted analysis were entered into a general linear model adjusted for maternal age, age at which education ceased, milk intake, smoking status (non- or ex-smoker), dairy products, and consumption of Brazil nuts.

The majority of women in the study (*n* = 222; 97%) were not taking a supplement that contained iodine, largely because most prenatal supplements that contain selenium (the use of which was an exclusion criterion) also contained iodine. Although median values tended to be higher, the iodine-to-creatinine ratio was not significantly different in the few iodine-containing supplement users at 12 (*n* = 6, *P* = 0.39), 20 (*n* = 6, *P* = 0.89), or 35 (*n* = 5, *P* = 0.12) wk of gestation.

Only 6% of women (*n* = 13) reported using iodized salt, and there was no significant difference in iodine-to-creatinine ratio between users and nonusers at 12 wk of gestation (*P* = 0.28). Of the dietary components investigated, only milk was significantly associated with iodine-to-creatinine ratio at 12 wk of gestation (Table 2), even in the adjustment model [i.e., when adjusted for maternal age, education, smoking status (non- or ex-smoker), and dairy products]. Iodine status was higher in those who reported consuming >1 portion of dairy products/d, but the differences were not significant (Table 2). Consumers of Brazil nuts tended to have higher iodine status than nonconsumers (*P* = 0.07), but the association was not significant in the adjusted model (Table 2).

We explored the effect of gestation on iodine-to-creatinine ratio using a linear mixed model in 219 subjects with a full set of covariates (**Table 3**). Five variables in the model were significantly related to the iodine-to-creatinine ratio, and these were as follows: BMI at 12 wk, maternal age, season, gestational week, and milk intake. Three 2-way interactions were significantly (at the 5% level) related to the iodine-to-creatinine ratio: gestational week and season (*P* = 0.026), gestational week and maternal age (*P* = 0.008), and maternal age and season (*P* = 0.047). The interaction between milk intake and season was significant at the 10% level (*P* = 0.074).

**TABLE 3:** Linear mixed model exploring the effect of week of gestation, season, and milk intake on iodine-to-creatinine ratio^1^

Season^2^	Geometric mean ratio (95% CI)^3^	*P*-interaction^4^	Adjusted *P*^5^
One-week increase in gestation			
Summer	1.040 (1.002, 1.079)	0.026	NA
Winter	1.050 (1.021, 1.080)		
Daily milk intake			
<140 mL			
Summer	1.000 (Ref)	0.56	1.00
Winter	1.040 (0.909, 1.192)		
140–280 mL			
Summer	1.000 (Ref)	0.16	1.00
Winter	1.086 (0.968, 1.219)		
>280 mL			
Summer	1.000 (Ref)	0.0003	0.0039
Winter	1.270 (1.119, 1.442)		

1 NA, not applicable; Ref, reference.

2 Summer: May–October; winter: November–April.

3 Exponential of β from the linear mixed model, controlling for the effect of BMI (at 12 wk), maternal age, season, and milk intake as well as first-order interactions.

4 *P* value for the difference in slope (summer to winter).

5 Bonferroni correction was applied for 15 pairwise comparisons between season and milk intake; only seasonal comparisons within the same milk intake category are shown.

Gestational week at which the urine was collected was significantly associated with iodine-to-creatinine ratio (*P* < 0.001). The model indicated a linear effect of gestational week on the iodine-to-creatinine ratio. There was a significant season-by-gestational week interaction (*P* = 0.026), such that the profile in winter increased more steeply than in summer (Table 3). In winter, the iodine-to-creatinine ratio increased 1.05 times per 1-wk increase, whereas in summer the increase was smaller, at 1.04 (Table 3). When extrapolating these values over 4 wk, the iodine-to-creatinine ratio increases by 1.21 times in winter (i.e., 1.05^4^) and 1.17 times in summer (1.04^4^). Over the course of a trimester (i.e., 12 wk), the increase would be 1.80 times in winter and 1.60 times in summer.

The interaction between season and milk intake on iodine-to-creatinine ratio was only significant in the group of women who reported a milk intake of >280 mL (*P* = 0.0003) (Table 3). The iodine-to-creatinine ratio was 1.27 times higher in winter than in summer in the group consuming >280 mL, which suggested a large effect of season on those who consume the most milk. There was no significant 3-way interaction between gestation, milk intake, and season (*P* = 0.34).

In the first and second trimesters, 36% (*n* = 83) and 35% (*n* = 77) of urine samples, respectively, were classified as dilute [creatinine concentration <0.3 g/L (25)], whereas a lower percentage (19%; *n* = 41) were dilute in the third trimester; this may be a result of women at the 12- and 20-wk visits attending scan appointments with a full bladder. In a sensitivity analysis, we excluded dilute urine samples to evaluate whether the increase in iodine status across gestation was a result of dilution bias. The effect of gestational week remained significant (*P* = 0.03), but the interaction between season and gestational week was significant only at the 10% level (*P* = 0.08 vs. *P* = 0.026 in the whole group).

## DISCUSSION

Our study confirmed our hypothesis that this cohort of UK pregnant women would be mildly-to-moderately iodine deficient. Importantly, because the women were classified as iodine deficient in each trimester, we have provided the first evidence that UK women treated as a group may be iodine deficient throughout gestation. The 3 measures of iodine-to-creatinine ratio were only moderately correlated, which suggested that a measure in 1 trimester will give minimal information on status at other stages of pregnancy. This is perhaps to be expected, given the day-to-day variability in iodine intake that influences iodine excretion, but it may also reflect a dilution bias, because urine samples at 35 wk were not as dilute as those at 12 and 20 wk.

Oxford historically had reports of goiter and thyroid enlargement in the 1930s and 1940s in both children (27) and young women (28), which persisted until the 1950s (29). Our finding of deficiency in the present day supports evidence of iodine deficiency in pregnancy in other parts of the United Kingdom, including Cardiff (7) and the North East (6), South East (8), and South West (2) regions. Women in SPRINT were largely reliant on diet alone, because just 3% were taking an iodine-containing supplement. Our findings suggest that these women need either specific advice on how to increase iodine intake during pregnancy or may require an iodine-containing supplement [although not kelp (30)] to meet requirements. In view of the fact that we previously found adverse associations between urinary iodine status in pregnancy and child cognition (2), the results from the present study are of considerable public health concern.

The UIC values were considerably lower than those of the iodine-to-creatinine ratio and were lower than those in other UK studies, including our own (8). This is probably because women were advised have a full bladder for their 12-wk ultrasound scan (when urine was collected) and thus samples were dilute. As a result, the iodine-to-creatinine ratio is likely to be more meaningful. Our findings show the importance of creatinine adjustment of UIC in studies that recruit women at their ultrasound appointment, because a methodologic bias from the collection of dilute samples may lead to an overestimation of iodine deficiency.

As in other UK studies (5, 8, 10), we found that milk intake was positively associated with iodine status. Interestingly, the effect of season was most pronounced in those women who consumed >280 mL milk/d. This is unsurprising because the reason that iodine status is affected by season is because of the higher iodine content of winter milk; hence, season is likely to have a minimal influence with low milk consumption.

Our results did not support our hypothesis that any change in iodine excretion during pregnancy would mostly be explained by season. We found an independent effect of gestation, but season modified this relation, with a steeper increase in winter than in summer. The results suggest that for a 1-wk increase, the iodine-to-creatinine ratio increases by 5% in winter and by 4% in summer.

The reason for the increase in iodine-to-creatinine ratio with advancing pregnancy is not clear, nor is the effect modification by season. The changes may reflect underlying physiologic changes that occur during pregnancy. However, changes in renal iodine loss, placental storage of iodine (31), or iodine transfer to the fetus are neither well characterized nor understood. In early pregnancy, there are high demands for thyroid hormone production, at least partly because human chorionic gonadotropin stimulates the thyroid (11), which is likely to result in greater thyroidal uptake of iodine, reducing the proportion excreted into the urine. This could explain why urinary iodine excretion was lower in early pregnancy than in later pregnancy, particularly given that the women were iodine deficient and would probably have had low thyroidal iodine stores. Alternatively, the increase in iodine excretion across gestation may represent a residual dilution bias, because samples at 12 and 20 wk were more dilute than those at 35 wk. Although we used the iodine-to-creatinine ratio to overcome the issue of dilution, this correction may not be as effective when samples are extremely dilute. When we excluded dilute samples, there was still a significant association with gestational week and a tendency for interaction between season and gestational week, but we cannot be certain that we effectively removed the dilution effect. The most likely explanation for the increase in iodine excretion with gestation is that dietary iodine intake, particularly from dairy products, increases as pregnancy progresses. In support of this hypothesis, the Southampton Women’s Survey found that intake of milk and dairy products increased from early to late pregnancy (32). If this were the case in our study, it might explain the observed seasonal differences.

To our knowledge, there are just 6 studies that used measures of iodine-to-creatinine ratio (as opposed to UIC) across pregnancy, and the results are conflicting. Studies reported both an increase (12, 33, 34) and a decrease (35–37) with advancing pregnancy. Results of the studies that evaluated the change in UIC across pregnancy are also contradictory, with data from 7 regions suggesting an increase (34, 36, 38–42), those from another 7 regions finding a decrease (35, 36, 43–46), and 10 studies finding no change (12, 33, 47–54). Those that found an increase (as in our study) were in iodine-deficient women and included both longitudinal (38–40) and cross-sectional (34, 36, 41, 42) studies. However, in at least 3 studies, the increase was attributed to an increase in supplement use in later pregnancy (38, 41, 55).

Our study has several strengths. For example, women were recruited year-round, which reduces the seasonal bias noted in another UK study (8). The design was suited to evaluating gestational changes in iodine excretion because we had repeated measures in the same individual, whereas many other studies explored differences in a cross-sectional study design (34, 36, 41, 42). In addition, women were excluded if they took a supplement containing selenium and therefore by default were largely not taking an iodine-containing supplement. Hence, in contrast to other studies (38, 41, 55), we were uniquely able to observe the underlying changes in iodine excretion largely without a background of supplement taking. However, our study also has several limitations. First, women were recruited to a trial, which may have introduced selection bias (e.g., those with higher socioeconomic status may have volunteered); hence, results may not be generalizable to the UK pregnant population. Second, we had no data on dietary change during pregnancy that might shed light on the increase in iodine excretion. Third, we reported change in iodine excretion in relation to the gestational week of urine sample collection, but as a result of the study design, samples were clustered around 12, 20, and 35 wk; we have no data before 9 wk or for every week of gestation. Fourth, we used a crude estimate of season as a proxy for milk-iodine concentration and this may not have accurately reflected differences. Finally, we recognize that a single spot-urine sample has limited use for individual iodine status assessment, and the day-to-day variation may explain some of the variability in the change across gestation; at least 10 repeat samples are required at the individual level for iodine status assessment (22). A repeat urine sample, collected from a subset of women, in each trimester would have given a better estimate of usual intake for the group (by correcting for intraindividual variation and adjusting the intake distribution to the mean) (56); unfortunately, repeat measures within a trimester were not available.

In conclusion, this study adds to the increasing evidence that pregnant women in the United Kingdom are mildly-to-moderately iodine deficient, particularly those not taking an iodine-containing supplement. Our finding of an increase in iodine status across gestation may not be generalizable to populations with different iodine status to ours and merits further investigation. Future studies should collect data more frequently (i.e., monthly) and account for underlying changes in season, particularly in countries in which a high proportion of iodine intake comes from dairy products. If our results are confirmed, trimester-specific criteria for classifying iodine status may be required.

## Footnotes

4 Abbreviations used: EAR, Estimated Average Requirement; FFQ, food-frequency questionnaire; SPRINT, Selenium in Pregnancy INTervention; UIC, urinary iodine concentration; UK, United Kingdom.
